# Dysmenorrhea increased the risk of postpartum depression in Chinese Han parturients

**DOI:** 10.1038/s41598-019-53059-8

**Published:** 2019-11-12

**Authors:** Liping Meng, Jianmei Li, Yuli Cheng, Tingting Wei, Yukai Du, Songxu Peng

**Affiliations:** 1 Department of Maternal and Child Health, School of Public Health, Tongji Medical College, Huazhong University of Science & Technology, Wuhan, Hubei China; 2 Department of Public Health, Bao an Maternal and Child Health Hospital, Jinan University, Shenzhen, Guodong China; 3 Department of Maternal and Child Health, Xiangya School of Public Health, Central South University, Changsha, Hunan China

**Keywords:** Depression, Risk factors

## Abstract

Several studies have shown that dysmenorrhea increased the risk of depression. However, the association between dysmenorrhea and postpartum depression (PPD) is unclear. The purpose of this study is to evaluate the effects of dysmenorrhea on the development of PPD among Chinese women. A case-control study was performed on parturients who delivered from January 1, 2016, to December 31, 2016, at Bao an Maternal and Child Health Hospital in Shenzhen, China. The Edinburgh Postnatal Depression Scale (EPDS) was used to screen for maternal postpartum depression. Logistic regression models were used to examine the association between dysmenorrhea and the risk of PPD. A total of 360 women including 120 cases and 240 controls were enrolled. Our study showed that parturients with PPD had a higher percentage of dysmenorrhea than women without PPD (64.2% *vs* 47.9%, *P* = 0.004). In univariate analysis, we observed that dysmenorrhea increased the risk for PPD (OR = 1.95; 95% CI: 1.24–3.06; *P* = 0.004). In the fully adjusted model, dysmenorrhea was still significantly associated with an increased risk of PPD (OR = 2.45; 95% CI: 1.36–4.54; *P* = 0.003). Our data confirmed that dysmenorrhea may be a risk factor for PPD. Therefore, screening for postpartum depression should be considered in parturients with a history of dysmenorrhea.

## Introduction

Postpartum depression (PPD) is a disabling but treatable maternal mental disorder that affects 0.5–60% of women worldwide and leads to substantial morbidity and mortality in mothers and children^1,2^. Mothers who suffer from PPD are also more likely to develop depression in the future^3,4^. PPD is often associated with a high risk of suicide, poor marital relationships, infanticide, and poor child development^5–8^. Accumulative evidence suggests that domestic violence, history of depression, stressful life events, marital dissatisfaction, lack of emotional or social support, anger experience and expression, and foetal or neonatal health problems increase the risk of developing PPD^9–13^. However, many risk factors remain unknown or poorly understood.

Dysmenorrhea, or menstrual pain, is a severe, painful, cramping sensation in the lower abdomen that is often accompanied by other symptoms, such as sweating, headaches, nausea, vomiting, diarrhoea, and tremulousness, all occurring just before or during menses^14^. Dysmenorrhea is also the most common gynaecological complaint and has a major impact on quality of life, work productivity, and health care utilization^15–17^. Several studies have indicated that dysmenorrhea is strongly linked with depression among adolescents^18–21^. A published study conducted by Bahrami *et al*. found that individuals in the dysmenorrhea group had significantly higher depression scores than normal controls^22^. Similar results were observed in Balık’s study, which concluded that adolescent girls with dysmenorrhea have an increased risk of depression^19^. PPD and major depression share many common risk factors unrelated to pregnancy, such as history of depression, domestic violence, stressful life events, emotional stress, and poor socioeconomic status. However, it is unclear whether dysmenorrhea can also affect the development of PPD. Hence, we hypothesized that dysmenorrhea is related to the development of PPD and increases the risk of PPD.

Based on above reasons, we performed this case-control study to investigate the possible association between dysmenorrhea and the risk of PPD 6 weeks after childbirth in a hospital-based sample of Chinese women.

## Results

### Demographic, socioeconomic, and pregnancy-related characteristics

Originally, 136 PPD cases and 272 controls were invited to participate this study, but only 120 cases and 240 controls were enrolled (a response rate of 88.2%) and completed the questionnaires. The socio-demographic characteristics and pregnancy-related factors of the two groups are presented in Table 1. Between the two groups, some demographic characteristics such as age at birth, education level, employment status, BMI, or family monthly income per capita were not significantly different. However, the proportion of primipara in the case group (68.3% *vs* 56.3%, *P* = 0.027) was higher than that in the control group.

**Table 1:** Maternal and neonatal characteristics according to the PPD status of women.

Characteristics	PPD		*P*-value
	Yes (n = 120)	No (n = 240)	
Age at birth (years)	29.04 ± 4.29	29.45 ± 4.30	0.391
**Education level**			**0.800**
High school or less	42 (35.0%)	81 (33.8%)	
College	31 (25.8%)	70 (29.2%)	
Graduate school	47 (39.2%)	89 (37.1%)	
**Employment status**			**0.104**
Full-time employed	80 (66.7%)	177 (73.8%)	
Self-employed	10 (8.3%)	24 (10.0%)	
Unemployed	15 (12.5%)	26 (10.8%)	
Other	15 (12.5%)	13 (5.4%)	
BMI	22.48 ± 3.84	22.13 ± 2.53	0.382
Primipara	82 (68.3%)	135 (56.3%)	**0.027**
**Family monthly income per capita**			**0.321**
<5000	43 (35.8%)	68 (28.3%)	
5000–10,000	42 (35.0%)	89 (37.1%)	
>10,000	35 (29.2%)	83 (34.6%)	
Depression during pregnancy	42 (35.0%)	7 (2.9%)	<**0.001**
Anxiety during pregnancy	66 (55.0%)	33 (13.8%)	<**0.001**
Stressful life events	21 (17.5%)	12 (5.0%)	<**0.001**
Planned pregnancy	68 (56.7%)	149 (62.1%)	0.322
Caesarean delivery	44 (36.7%)	92 (38.3%)	0.758
Preterm birth	8 (6.7%)	8 (3.3%)	0.148
Low birth weight	12 (10.0%)	3 (1.3%)	<**0.001**
Male infant	68 (56.7%)	116 (48.3%)	0.136
**Breastfeeding status at 6 weeks**			**0.591**
Exclusive	70 (58.3%)	130 (54.2%)	
Partial	40 (33.3%)	93 (38.7%)	
Formula only	10 (8.4%)	17 (7.1%)	
Dysmenorrhea	77 (64.2%)	115 (47.9%)	**0.004**

Women with PPD were significantly more likely to experience depression (35.0% *vs* 2.9%, *P* < 0.001) and anxiety (55.0% *vs* 13.8%, *P* < 0.001) during pregnancy than other women. The patients with PPD were also more likely to have a stressful life event experience (17.5% *vs* 5.0%, *P* < 0.001) compared with those in the control group. Additionally, there was a significantly higher incidence of low birth weight in the PPD patients (10.0% *vs* 1.3%, *P* < 0.001). In the case group, 77 (64.2%) women also reported a history of dysmenorrhea, which was significantly higher than the corresponding figure in the control group (64.2% *vs* 47.9%, *P* = 0.004). However, there was no significant difference in the other pregnancy-related factors, including planned pregnancy, mode of delivery, preterm birth, infant gender, or breastfeeding status, between the case group and the control group at 6 weeks.

### SSRS scores

As shown in Table 2, compared with the control group, PPD patients scored significantly lower on the objective support (8.38 ± 2.97 *vs* 9.68 ± 2.87, respectively; *P* < 0.001), subjective support (20.82 ± 4.41 *vs* 24.20 ± 4.02, respectively; *P* < 0.001), and support usage (7.03 ± 1.62 *vs* 8.17 ± 3.67, respectively; *P* < 0.001) subscales.

**Table 2:** Comparison of social support scores between women with and without PPD.

Characteristics	PPD		*P* value
	Yes	No	
Objective support	8.38 ± 2.97	9.68 ± 2.87	<0.001
Subjective support	20.82 ± 4.41	24.20 ± 4.02	<0.001
Support usage	7.03 ± 1.62	8.17 ± 3.67	<0.001

However, the significant differences in primipara did not pass the Bonferroni corrections (Bonferroni corrected *P* < 0.05/18 = 0.002).

### Multivariable logistic regression analysis for the association of dysmenorrhea and PPD

As shown in Table 3, dysmenorrhea was significantly associated with a higher risk of PPD in model 1 (OR = 1.95; 95% CI: 1.24–3.06; *P* = 0.004). After adjustment for other covariates including age, education level, BMI, employment status, parity, and family monthly income per capita, dysmenorrhea was still associated with an 85% increase in the risk of PPD (model 2: OR = 1.85; 95% CI: 1.16–2.95; *P* = 0.010). Considering the effects of pregnancy-related risk factors on the outcome variable, we further added those variables into the multivariable logistic regression model. As a result, the association between dysmenorrhea and the risk of PPD remained significant (model 3: OR = 2.19; 95% CI: 1.25–3.85; *P* = 0.006). Finally, the social support scores including those of objective support, subjective support and support usage were included in the model as continuous variables on the basis of model 3. In the fully adjusted model, women with a history of dysmenorrhea had higher prevalence of PPD compared to women without a record of dysmenorrhea (model 4: OR = 2.45; 95% CI: 1.36–4.54; *P* = 0.003).

**Table 3:** Multivariate logistic regression analyses of the association between dysmenorrhea and PPD.

	OR	95% CI	*P*-value
Model 1	1.95	1.24–3.06	0.004
Model 2	1.85	1.16–2.95	0.010
Model 3	2.19	1.25–3.85	0.006
Model 4	2.45	1.36–4.54	0.003

Abbreviation: OR, odds ratio; CI, confidence interval.

Model 1: unadjusted.

Model 2: adjusted for age, education level, BMI, employment status, parity, and family monthly income per capita.

Model 3: adjusted for covariates in model 2 and depression during pregnancy, anxiety during pregnancy, stressful life events, planned pregnancy, mode of delivery, preterm birth, low birth weight, infant gender, and feeding method at 6 weeks.

Model 4: adjusted for covariates in model 3 and subjective support, objective support, and support usage.

## Discussion

To the best of our knowledge, this is the first study to investigate the association between dysmenorrhea and the risk of PPD in a Chinese Han population. Moreover, our study found a higher risk of PPD in women with dysmenorrhea.

Little previous evidence was available on the association between dysmenorrhea and the risk of PPD; however, a number of previous studies reported that dysmenorrhea was associated with a high risk of depression^18–22^. One previous study indicated that women who had a history of dysmenorrhea were more likely to suffer from depression or had a higher score for depression compared with women without menstrual pain^18^. Additionally, Coleman and his colleagues also found that severe menstrual pain was an important risk factor strongly associated with depression^20^. Our study suggests that parturient women with dysmenorrhea have a high risk of PPD; this result is consistent with the findings from a Japanese study that included 11,341 PPD patients and 71,148 controls^23^. Their study demonstrated that parturients with dysmenorrhea had 1.13 times higher risk of developing PPD (OR, 1.13; 95% CI: 1.06–1.21), after adjustment for socio-demographic factors, health behavioural factors, psychiatric illness history, psychosocial factors, obstetrical factors and birth outcome factors^23^. However, an American study by Swenson showed that there was no significant difference in the distribution of dysmenorrhea between women with and without a positive PPD test^24^. Possible reasons for this difference include the different criteria used for the diagnosis of PPD and the different study designs adopted by investigators.

Based on the underlying cause of developing dysmenorrhea, dysmenorrhea is commonly classified into two categories: primary and secondary dysmenorrhea. Primary dysmenorrhea, characterized by menstrual pain without organic disease, is considered to be caused by excess secretion of prostaglandin associated with changes in oestrogen and progesterone levels^25^. A great number of studies have reported that changes in ovarian hormones are associated with a high risk of depression^26,27^. Preclinical and clinical studies have shown that oestrogen modulates the expression of genes that code for tryptophan hydroxylase, the serotonin transporter, and the 5-HT1a auto-receptor^28,29^. Hence, the fluctuation of oestrogen could cause an alteration in serotonin neurotransmission, which then leads to mood disorders^26^. Binding to receptors that are localized in the region of the brain that is involved emotional and cognitive regulation, progesterone also regulates neurotransmitter synthesis, release, and transport^30,31^, and further causes dysfunctional mood regulation^32^. In addition to the effects of hormones, in recent years, many studies have confirmed that depressive disorders are associated with increased concentrations of many pro-inflammatory cytokines, including tumour necrosis factor α (TNF-α) and interleukins (ILs)^33–36^. These pro-inflammatory cytokines lead to a deficit in serotonin and melatonin through the kynurenine pathway^35^, which is considered one of the main reasons for depression. Furthermore, a study by Ma and colleagues demonstrated that the genes encoding pro-inflammatory cytokines (IL1B, TNF, IL6, and IL8) were up-regulated in peripheral blood mononuclear cells (PBMCs) of primary dysmenorrhoeic young women during the menstrual phase^37^. Therefore, dysmenorrhea may lead to the development of PPD though an increase in pro-inflammatory cytokines.

Secondary dysmenorrhea is menstrual pain associated with an identifiable disease. Endometriosis is the most common cause of secondary dysmenorrhea, and its development is likely dependent on sex hormones, especially oestrogen^25^. A high level of localized oestrogen is frequently observed in endometriosis patients^38^. Clinical studies demonstrate that oestrogen can affect serotonin synthesis, receptor production, or degradation^28,29^, and the effects of oestrogen on serotonin are commonly considered to be important for mood.

This study is the first to investigate the relationship between dysmenorrhea and PPD among the Chinese Han population. Another strength of this research is that the potential confounding variables including socio-demographic factors and pregnancy-related risk factors, were considered in the multiple regression model. There is no denying that the present study has several limitations. First, a case-control study design cannot assess direct causality between dysmenorrhea and PPD. Second, we were not able to separate secondary from primary dysmenorrhea because we lacked information on conditions leading to secondary dysmenorrhea. Third, the EPDS is just a screening tool and not a diagnostic tool, although the measurement of PPD with the EPDS has been widely used and validated in China.

In conclusion, our study indicated that dysmenorrhea was independently associated with PPD. The risk of PPD was clearly higher in women who had history of dysmenorrhea. Therefore, screening for postpartum depression should be considered for postpartum women with persistent dysmenorrhea.

## Methods

### Study design and population

The present study was a case-control study carried out from January 1, 2016, to December 31, 2016, at Bao an Maternal and Child Health Hospital, Shenzhen, China. All women delivered in this hospital, were more than 18 years old, and were screened for postpartum depression using the Edinburgh Postnatal Depression Scale (EPDS), at a routine follow-up appointment 6 weeks after delivery. As described in a previous report, women with an EPDS score ≥10 were considered to have postpartum depression and were eligible for joining the case group^39^. PPD patients were consecutively recruited from postnatal obstetric clinics. Inclusion criteria for the case group were having a confirmed diagnosis of clinical PPD using EPDS, agreeing to participate in this survey and signing an informed consent form. However, those with severe physical illness (including infectious disease, cardiovascular disease, gastrointestinal disorder, endocrine disorder, urinary disease, and benign or malignant tumours), disability, psychotic disorders, or substance abuse problems were excluded from the study. The intent of this study was to have 2 controls for every case, and controls were randomly selected from patients who attended postnatal obstetrics clinics. Only those with an EPDS score <10 were invited to participate and assigned to the control group. The inclusion/exclusion criteria for the control group were almost identical to that of the case group, besides the absence of PPD.

### Data collection and procedures

Well-qualified investigators were assigned to collect the data for this study. Some obstetricians and nurses of Bao an Maternal and Child Health Hospital served as the investigators of this study. All investigators participated in the training conducted by the first author before starting data collection. After training, the investigators carried out face-to-face interviews with the participants using a structured questionnaire, which was specially designed based on existing the literature and expert consultation. The questionnaire was used to collect data regarding socio-demographic characteristics (age, education level, employment status, height, weight, and family monthly income per capita), and the pregnancy-related risk factors (planned pregnancy, depression and anxiety during pregnancy, stressful life events, mode of delivery, gestational weeks, birth weight, gender of foetus, and infant feeding method). The accuracy of these data was then checked with the participants’ medical records. In the interviews, women were also asked whether they had a history of dysmenorrhea.

### Measures

#### Social support

The Chinese version of the Social Support Rating Scale (SSRS) developed by Xiao ShuiYuan in 1994, was used to assess the individuals’ social support status. The SSRS consists of 10 items involving three dimensions, namely, objective support (3 items), subjective support (4 items), and support utilization (3 items)^40^. The total score on the SSRS ranges from 12 to 66, including objective support (range: 1–22), subjective support (range: 8–32) and support usage (range: 3–12). Higher SSRS scores indicate better social support. This tool has been proven to have good reliability and validity. Cronbach’s alpha coefficients for the total scale and subscales ranged from 0.825 to 0.896^40,41^.

#### Postpartum depression

The Edinburgh Postnatal Depression Scale (EPDS) has been widely used to evaluate maternal postpartum depression. The EPDS has 10 items, and each item is scored on a 4-point scale ranging from 0 to 3. Total scores range from 0 to 30, with higher scores reflecting an increased risk of PPD. The validity and reliability of the Chinese version of the EPDS have been reported, with a sensitivity of 0.82 and a specificity of 0.86^42^. In the present study, a total score of 10 or higher was defined as PPD, consistent with previous research^39^.

### Ethics statement

All study procedures were approved by the Institutional Review Board of Tongji Medical College, Huazhong University of Science and Technology and Bao an Maternal and Child Health Hospital, Jinan University. The methodology was carried out in accordance with the approved guidelines. Written informed consent was obtained from all participants.

### Statistical analysis

Categorical data are presented as numbers and percentages (%), continuous variables are described as the mean ± standard deviation (SD). Student’s t-tests were used to test continuous variables and chi-square tests were used to compare categorical data. We used Bonferroni corrections to adjust for multiple testing. The adjusted odds ratios (ORs) with 95% confidence intervals (95% CI) were used to measure the independent association between PPD development and dysmenorrhea. The statistical power to detect the difference in dysmenorrhea between the case group and control group was calculated by Power and Sample Size Calculation v3.1.2. We calculated that the power for our sample size to detect an OR of 1.50 was 0.981. The significance level was set at 5%, and all tests were two-sided. All analyses were performed using SPSS software version 18.0 (SPSS, Chicago, IL, USA).

## Data Availability

All data generated or analysed during this study are included in this published article.
